# Personal Mention

**Published:** 1915-12

**Authors:** 


### Personal Mention.

Dr. W. B. Collins, State Health Officer, attended the funeral of Dr. L. M. Stroud, of Terrell.

Dr. J. W. Mickle, of Memphis, has moved to Colorado, Texas, where he will continue to practice medicine.

Dr. John N. Thomas, Superintendent of the Insane Asylum at Alexandria, La., visited in Kaufman the first part of the month.

Dr. Edgar Gilcreest, of Gainesville, Texas, has returned to the United States from Rome. Dr. Gilcreest has been in Europe for several months.

Dr. John C. Bell, President of the Board of Health of Memphis, Tennessee, spent a few days in Loraine, Texas, as a guest of Dr. J. B. Sparks.

Dr. M. A. King has resigned his position as mill physician for the Foster Lumber Company of Beaumont, and his place has been filled by Dr. W. H. Jones, of Colmesneil.

Dr. Hugh F. Young, of Baltimore, Professor of Surgery in the Johns Hopkins University, who attended the meeting of the Southern Medical Association, spent a few days in Sherman.

Dr. Frank Nugent, a dentist of Fort Worth, has moved to Paducah, Texas.

Dr. C. H. Reagan, of Beeville, has returned from Rochester, Minn., where he went to be with his brother during an operation for appendicitis.

While on his way to attend the meeting of the Southern Medical Association at Dallas, Dr. J. A. Daniels broke his wrist cranking his car.

Miss Eula Powell, daughter of Dr. and Mrs. Powell, of Terrell, and Dr. H. W. G. Shytles, of Fort Worth, were married in Terrell on November 23d.

Dr. O. S. Hodges, who has been in New York and Philadelphia doing hospital work for the past five weeks, has returned to his home in Beaumont.

Dr. R. W. McFerran has returned from New Orleans, where he took a course of lectures. Dr. McFerran lives in Childress, Texas.

Dr. C. J. Parrish, of Greenwood, has formed a partnership with Dr. J. J. Ingram, and will move to Decatur about the first of January.

Dr. Chas. M. McMillan and wife, of Plantersville, Texas, have left for a six weeks' visit to California and other points on the coast.

The election of Dr. Holman Taylor, of Fort Worth, as First Vice-President of the Southern Medical Association, was another victory for Texas and Texans. Dr. Taylor was worthy of this honor, and his helpful advice will no doubt prove very beneficial. He has been an untiring worker in the field of organized medicine for many years, and no man in this State is better known.

---

### Dr. C. B. Spates of Des Moines Exhibits Patients and Describes "Intra-Venous" Treatment.

Before the Rotary Club, Dr. C. B. Spates, of Des Moines, declared without qualification that a cure for the white plague—tuberculosis—has been discovered.

He brought four of his patients before the Rotarians to substantiate his statement. He pronounced two of them cured of the disease and the other two, now undergoing his new treatment, almost cured.

Dr. Spates explained the treatment. He called it the "Intravenous treatment" It is a compound of drugs injected into the veins of the arm of a patient. Dr. Spates declares he makes no secret of the drugs and medicines he uses in making the fluid. He says many doctors know of it already.

Dr. Spates says the intra-venous treatment will cure tuberculosis in from six to fifteen weeks. It requires from three to five injections, he told the Rotarians. The injections are made from two or three weeks apart.

---

### Helpful Hints for the Busy Doctor.

The Storm Binder and Abdominal Supporter.—The problem of securing a proper and efficient abdominal support during pregnancy and after confinement as well as after laparotomies is an important one, and has in recent years been extended considerably, since the importance of relieving all varieties of enteroptosis by mechanical support has been realized. The treatment of enteroptosis, of floating kidney and even of cholelithiasis (according to Achilles Rose) by a well fitting abdominal support has been successful in a large number of cases. It is, however, indispensable that the support should not only be properly adjusted and should hold the prolapsed viscera in place, but it must also be free from discomfort, it must be washable, durable in quality and moderate in price.

All these requirements are unusually well met in the Binder and Abdominal Supporter made in many varieties and for all conceivable pur-

